# Empowering local researchers toward using local data to identify local health priorities: A reflection on three cohorts of the course “Write your own paper using Demographic and Health Survey data on reproductive and child health”

**DOI:** 10.7189/jogh.13.01004

**Published:** 2023-12-15

**Authors:** Lenka Beňová

**Affiliations:** Department of Public Health, Institute of Tropical Medicine, Antwerp, Belgium

The Demographic and Health Surveys (DHS) represent a tradition of repeated, rich, rigorous, and informative health and demographic data for low- and middle-income countries (LMICs) [1]. While these data are available and commonly used in research globally, they are under-used in guiding scientific inquiry and health-sector decisions by researchers, students, and policymakers in the countries where they are collected. Laudable efforts have been made to make the survey findings more accessible through, for example, survey and analytical reports [2], the StatCompiler tool [3], and targeted workshops, mostly facilitated by the ICF, the agency responsible for conducting the DHS [4]. However, delving beyond standard indicators and stratifications available on DHS reports and examining associations requires a researcher to possess several advanced competencies to correctly and meaningfully analyse these invaluable secondary data, write a scientific manuscript, and disseminate findings to inform local policy and action.

At the Institute of Tropical Medicine (ITM) in Antwerp, Belgium, part of our mission is to share capacity through education and training, among other activities. Since 2020, we have organised three annual editions of a short course aiming to build capacity for conducting quantitative analysis of DHS data and to contribute to expanding the country-level capacity to further use and impart such skills [5]. This course aims to support researchers in analysing DHS data, focusing on a wide range of topics spanning sexual, reproductive, maternal, newborn, child, and adolescent health. Its objective is to prepare an advanced draft of a publishable paper and to submit the manuscript to a scientific journal. This course is primarily, but not exclusively, aimed at early to mid-career health researchers from LMICs (partial and full scholarships are available), including master’s degree-level graduates and PhD students. The application process involves submitting a short proposal with a specific research question answerable by analysing DHS data. Participants must have a good command of a statistical software package (the course can support users of Stata, R, and SPSS) and a working knowledge of statistical methods commensurate with the proposed analysis plan. This course uniquely provides a learning environment consisting of a small group (8-15 participants per cohort), intensive personal support from an expert (“coach”), and the use of adult learning methods to support participants to further develop their research question into a detailed analysis plan (specifically, approaches from transformative learning theory and experiential learning), resulting in a publishable manuscript [6]. Participants are exposed to each others’ topics and questions during the course to expand their learning and perspectives.

## OBJECTIVE

The objective of this commentary is twofold: to reflect on three years of experience running this course and to introduce a series of papers written by participants in the 2022 cohort.

## EXPERIENCE FROM THREE COURSE COHORTS

We designed an annual, blended course composed of 13 weeks online followed by one week in-person at ITM’s Antwerp campus. It was first held in 2020 in a fully online mode due to the coronavirus disease 2019 (COVID-19) pandemic, which negatively interacted with the online modality and significantly disrupted the participants’ ability to allocate sufficient time to their analysis, partly due to competing demands on health workers and researchers globally. The fully online learning mode also limited the extent of peer exchange and support. Hence, we chose not to organise the course in 2021, when COVID-19 restrictions were still in place. Two blended courses were organized since then, in 2022 and 2023. Overall, 35 participants from 22 countries completed the three course editions (24 from Africa, three from Asia, two from the Middle East, four from Europe and one from Latin America); 18 of these participants benefited from a full or partial scholarship.

The unique characteristics of the course can be summarized along three axes: selectivity, intensity and multi-disciplinarity. First, the course is targeted at a narrow profile of participants – those who have the appropriate statistical competencies for the analysis they are pursuing, but who need support with the specificity of DHS data analysis and paper-writing. We also take the relevance of the research question and the quality of the proposal into account for the selection. The acceptance rate is thus relatively low at 20%; some applicants are rejected because they already published analyses of DHS data and are therefore deemed overqualified. Second, intensity refers to the amount of guidance and support participants receive from the course team in general and their personal coach in particular. Each participant is matched with an experienced coach who supports them in finalising the research question, data management and analysis, and paper writing. Due to the in-depth nature of this involvement, one coach can guide a maximum of two participants. With such a high coach-to-participant ration, the organisation of this course is extremely resource-intensive, particularly in terms of identifying and engaging experts as coaches. Third, multi-disciplinarity refers to the overall range of support available to participants within the course from the various coaches and technical advisors (demographer, statistician, and geospatial modeller) regarding professional backgrounds, thematic areas, software, statistical methods, technical questions about the survey design and questionnaire, and experience with the process of publishing a scientific paper. During the face-to-face week, we also address more general points related to all participants, such as research ethics (for example, *P*-value “fishing”), choosing an appropriate journal, and writing a cover letter for paper submission.

## PAPER SERIES IN JOURNAL OF GLOBAL HEALTH REPORTS (JOGHR)

While the course benefits from a motivated group of participants, the process of finalising a manuscript beyond the formal 14-week course schedule can be challenging and unpredictable. The coaches remain involved in the capacity of co-authors depending on the participants’ preferences, but progress on writing and editing a paper can slow down when participants return to their daily responsibilities after the intense face-to-face week. Further, issues beyond the control of the participant, coach, and the course team can arise. This includes having to communicate and negotiate with a number of additional co-authors and supervisors (which can be a stressful task for an early-career researcher) and identifying funding for the open access fee (which the course at ITM cannot support). To assist with the timely completion and publication of papers of the participants on the 2022 course, we experimented with organising a formal paper series with the Journal of Global Health Reports. Participants were invited to submit to the Series and editors of the Journal committed to a timely peer-review and lower open access fee for participants of the course.

Between September 2022 and August 2023, six of the 12 participants on the 2022 course published a paper in this Series. In addition, two participants published in other journals [7,8]. The six papers in the series cover topics ranging from sexual and reproductive health (contraceptive use) and maternal health (pregnancy and postpartum) to child health (breastfeeding, health-seeking behavior and malnutrition) (**Table 1**). The studies used one or multiple surveys in six countries (Cambodia, Ethiopia, Malawi, Nigeria, Sierra Leone and Tanzania). These papers not only contain rigorous, high-quality analyses, but also provide an excellent example of having achieved the course objective of concluding with timely, specific and locally relevant recommendations for further research and policy prioritisation.

**Table 1:** Description of the six papers in the JOGHR series resulting from the 2022 cohort in the ITM course “Write your own paper from the DHS on reproductive and child health”*

Author (year)	Objective	Study country, survey years used	Key findings and recommendations
Emmanuel Olal et al. (2023) [9]	To assess the trends of modern contraceptive use among adolescent girls aged 15-19 years in Sierra Leone in the period 2008-2019 and to explore how individual and contextual determinants of their use changed during this period.	Sierra Leone, three surveys (2008, 2013, 2019)	Modern contraceptive prevalence among adolescents in Sierra Leone increased from 5.9% in 2008 to 21.0% in 2019; but most of this increase occurred by 2013. Interventions to increase awareness and accessibility of modern contraceptives among adolescents in Sierra Leone are urgently needed, especially among adolescents without formal education. This might include affordable community- and school-based provision of contraception using adolescent-friendly services.
Amani Kikula et al. (2023) [10]	To describe trends over time in the distribution of BMI among Tanzanian women of reproductive age intending to conceive and to identify factors associated with high BMI (overweight and obese).	Tanzania, three surveys (2004, 2010, 2015/16)	Among Tanzanian women of reproductive age intending to conceive, the prevalence of overweight increased from 11.1% in 2004 to 15.8% in 2015/16 and the prevalence of obesity increased from 3.1% in 2004 to 8.0% in 2015/16. In the setting of Tanzania, where high BMI affects nearly one in four women of reproductive age who intend to conceive, it is urgent to develop and implement health-system strategies to prevent and address this burden.
Abdulaziz Hussen et al. (2023) [11]	To examine the trends of immediate postnatal care utilisation from 2011 to 2019 and to identify factors associated with immediate postnatal care utilisation.	Ethiopia, three surveys (2011, 2016, 2019)	The percentage of women who received immediate postnatal check increased from 6.4% in 2011 to 33.4% in 2019. Four or more antenatal care visits and caesarean delivery were factors positively associated with immediate postnatal care utilisation. Despite improvements in immediate postnatal care utilization between 2011 and 2019, the coverage is suboptimal and calls for context-specific efforts across the continuum of maternal healthcare services to improve the provision, utilisation, and quality of postnatal care.
Tope Olubodun et al. (2023) [12]	To examine the association between recent maternal experience of IPV and optimal breastfeeding of children aged 0 to 23 months.	Nigeria, one survey (2018)	31% of women experienced any IPV and 2.6% experiences all three forms (physical, psychological and sexual). Nearly one-third (31.7%) of babies <6 months of age and 70.4% of six to 23-month-olds were optimally breastfed. We found no significant association between experiencing any IPV and optimal breastfeeding for age. However, women who experienced all three forms of IPV were significantly less likely to optimally breastfeed their children than those with no IPV experience. Strategies to promote breastfeeding should consider the burden of IPV against breastfeeding women and seek to provide support for those who are victims.
Yusuf Salim et al. (2023) [13]	To identify factors associated with prompt health-seeking behaviour among caregivers of children under five with fever.	Malawi, one survey (2017 Malawi Malaria Indicator Survey)	40.1% of children under five years presented with fever in the two weeks before the survey, and one-third of these children were taken for treatment. Public health facilities are the most visited places for under five child treatment (68%), followed by private drug sellers/pharmacies (1%) and private/religious facilities (15%). Further qualitative research should inform the design of health education programs to raise awareness among caregivers of the importance of early treatment-seeking, regardless of child age.
Asuka Miyazaki et al. (2023) [14]	To assess complementary feeding practice and investigate its association with linear growth faltering among children aged 6 to 23 months among children aged six to 23 months.	Cambodia, one survey (2014)	Prevalence of age-appropriate complementary feeding practice in Cambodia was low: only 23% of children met all three criteria of minimum dietary diversity, minimum meal frequency, and age-appropriate breastfeeding. There was no association between food diversity and feeding frequency and child linear growth. Maternal and child nutrition educational interventions are needed to improve feeding and hygiene practices.

BMI – body mass index, IPV – intimate partner violence

*All mentioned first authors were course participants.

## WAY FORWARD

We seek to continually improve the delivery of this unique course and to ensure personalised support to the course participants. There is increasing demand to support analyses of surveys from multiple countries, which has important implications on the extent to which the course participants are able to derive context-specific, locally-relevant recommendations and actions. Such multi-country analyses are also often framed within a geospatial analysis perspective, which requires expertise in integrating additional data sets to study topics such as quality of care (e.g. Service Provision Surveys (SPAs)), location of health care facilities (publicly available data sets of health facilities such as the Pan-African database of public health facilities) [15], and infrastructure and atmospheric variation (e.g. road networks, temperature and precipitation). We need to carefully balance the technical expertise needed to support advanced analyses such as accessibility of health care and climate change-related disruptions with the ambition to have the majority of the coaches from LMICs. This appears possible – in 2020, all five coaches were from high-income countries; by 2023, five of the nine coaches were from LMICs, including two who had previously received training on the use of DHS by the ITM team. Further, we are in discussions with research institutions in LMICs to host the face-to-face week in the future to reduce the burden of travel and visa requirements on course participants who are predominantly from LMICs. We wish to accomplish this while ensuring that the scholarships and funding for the organization of the course continue and are well allocated.
